# Huxley's Logic and Life

**Published:** 1893-05-27

**Authors:** 


### The Hospital

AN INSTITUTIONAL JOURNAL OF

**Science, Medicine, Nursing, and Philanthropy.**

*For Hospitals, Asylums, Infirmaries, Dispensaries, Medical Practitioners, Students, Nurses, and the Charitable Public.*

VOL. XIV.—No. 348.] MAY 27, 1893.

### HUXLEY'S LOGIC AND LIFE.

LIFE is livelier than logic; and Mr. T. H. Huxley is a much more interesting subject than even his own philosophical sp culations. Many men, when considered apart from their work, are found to be not worth consideration. Not so Mr. Huxley As with Samuel Taylor Coleridge and Dr. Johnson his personality is the prime part of him. Last week Mr. Huxley delivered the Romanes Lecture at Oxford; and, in the presence of the most brilliant and ardent intellectual life of modern England and the world, essayed a criticism of man in the light of all past speculations.

What is the measure which Mr. Huxley takes cf man after a long life devoted with the utmost strenuousness to the scientific and philosophical study of man and his animal kindred? Mr. Huxley is pre-eminently honest and robust in mind, and almost defiantly fearless. From him we may look with confidence for honest, soundly reasoned, and modern opinions. What are those opinions at the present stage of his life? In our judgment this is not an unimportant question. Thinkers who are both honest and capable divide the guidance of the world among themselves, and always have done. In the long run it is the brain that rules, not the mere physical or even the moral energy, but the thinking and judging powers.

We must confess to some bewilderment at finding Mr. Huxley ranging himself, with full consciousness of his apparent inconsistency, on two sides of the philosophical battle at one and the same time. He is a cosmical evolutionist and an ethical progressist. Here it is in a sentence. "The practice of that which is ethically b st," says Mr. Huxley, "what we call goodness or virtue, involves a course of conduct which in all respects is opposed to that which leads to success in the cosmic struggle for existence. In place of ruthless self-assertion it demands self.restraint; in place of thrusting aside or treading down all competitors, it requires that the individual shall not merely respect but shall help his fellows; its influence is directed not so much to the survival of the fittest, as to the fitting of as many as possible to survive. It repudiates the gladiatorial theory of existence."

Now this we believe to be excellent morals and good science, but conspicuously deficient in insight and sound logic. The chief fallacy will be found in the too limited significance attached to the word "fittest." The influence of the practice of goodness, says Mr. Huxley, "is directed not so much to the survival of the fittest as to the fitting of as many as possible to survive." Here we join issue absolutely with Mr. Huxley, and our thesis is that the virtuous are the fit: the best are the fittest. How otherwise have virtue and goodness made gradual conquest of the world? If this be so, the "fittest" will continue to be the survivors. For we must first ask ourselves, Who are the best? and then we must ask ourselves, How and in what manner do they actually and potently survive? The best we hold to be the ablest, the justest, the most strenuous for the good and happiness of the race. But ableness, justness, and strenuousness are the most resolute of all the elements of survival. Let this definition of the best be duly considered. The merely amiablest are not the best: the most self-renunciatory are not the best; they may be good, but not the best. We take it for granted that Mr. Huxley has too hastily assumed these to be the best, and that on the authority of modern but lean and limited Christianity. But much consideration, devoted for a long time to this point, has led the writer to the conclusion that mere amiability and self-denial do not entitle a man to rank with the best, and do not, therefore, argue in him the highest degree of "surviving" endowment. Moreover, we think Christianity in both the life and the teachings of its founder is on our side of the argument, and insists that the actual best are the ablest, the justest, and the most strenuous for the good and happiness of the race.

Is our contention sound? Will it bear the severest critical examination? If it will, then there is no such real, deep, permanent, and inevitable antagonism between the cosmic process and the highest ethical ideals as Mr. Huxley insists upon. True, there is a very obtrusive appearance of such antagonism; and in his contention Mr. Huxley finds himself fighting under the banner of St. Paul, who reiterated in a variety of forms his contention that the "carnal mind" is "enmity against God." But a truer view seems to be that the first cause, the primal and eternal King and energiser, works through the ages with consistent uniformity, with uniformity at least of object and purpose, even if not at all times of evident and manifest process.

In a brief article like the present it is obvious that we can only indicate a position—conditions of space absolutely prohibit any attempt at reasoned justification. We think, however, that the position can be justified, though it may possibly require for its complete justification the doctrine of a future existence and of present probation. But even so, Mr. Huxley is far too profoundly read a man, and has far too much respect for the opinions of the many competent thinkers of past ages, to immediately reject the contention on that account Be that as it may, one cannot but feel that it is in many ways undesirable that philosophy should ask us to admit a rooted, permanent, inherent, and inevitable antagonism between the cosmic process and the ethical ideal. A more credible philosophy bids us by all possible means get rid of the theory of such an antagonism, if it can be logically and rationally got rid of.

But, speculation apart, it is profoundly interesting to see Mr. Huxley, at the age of nearly seventy, taking an immovable stand on the doctrine of the priceless and undying worth of virtue, and setting the intuitions and instincts of his consciousness higher in authority than the ablest elaborations of his reason. For that is what it really amounts to. "Ethical nature," he says, "may count upon having to reckon with a tenacious and powerful enemy so long as the world lasts. But, on the other hand, I see no limit to the extent to which intelligence and will, guided by sound principles of investigation and organised in common effort, may modify the conditions of existence. . . . And much may be done to change the nature of man himself." This last sentence should be printed in letters of gold by all teachers of morals and religion; nay, by all persons who understand humanity and desire its well-being. "Much may be done to change the nature of man himself." This is an ardent aspiration on the part of a man who has subjected every known theory of life to competent criticism, and who, in the final outcome, repudiates selfishness and the gladiator in favour of self-renunciation and the love of his fellow-man.

For practical purposes, physiology, in the person of its most distinguished modern professor, has arrived at the same conclusion as all great philosophies and all noble religions. That conclusion is that "On earth there is nothing great but man; and in man there is nothing great but mind." The cosmic process is almost irresistible, and appears to be irresistible in a non-ethical direction. The chief business of man, the noble conflict, according to Mr. Huxley, is to be virtuous, and, if need be, to oppose the cosmic process where it appears to be antagonistic to virtue, and to subdue that process, first within himself, and then in the external world, to the higher laws of justice, compassion, and mutual help. Have we any fault to find with this conclusion? None! It has been arrived at by intellectual methods—but not entirely. There has been, through all Mr. Huxley's life contests and experiences, an inner consciousness, endowed from birth with intuitions, potencies, and "streams of tendency making for righteousness," as Matthew Arnold called them; and the intellectual offspring of Mr. Huxley's mature age owns this mixed inherited and logical parentage. It is to the virtue of that mixed parentage that the offspring owes its innately noble and permanently enduring character.

